# Modulations of Neuroendocrine Stress Responses During Confinement in Antarctica and the Role of Hypobaric Hypoxia

**DOI:** 10.3389/fphys.2018.01647

**Published:** 2018-11-26

**Authors:** Claudia Strewe, Detlef Thieme, Carole Dangoisse, Barbara Fiedel, Floris van den Berg, Holger Bauer, Alex P. Salam, Petra Gössmann-Lang, Patrizia Campolongo, Dominique Moser, Roel Quintens, Marjan Moreels, Sarah Baatout, Eberhard Kohlberg, Gustav Schelling, Alexander Choukèr, Matthias Feuerecker

**Affiliations:** ^1^Laboratory of Translational Research “Stress and Immunity”, Department of Anaesthesiology, University Hospital, LMU Munich, Munich, Germany; ^2^Institute of Doping Analysis and Sports Biochemistry, Dresden, Germany; ^3^IPEV/PNRA-ESA Antarctic Program, Brest, Antarctica; ^4^Alfred-Wegener-Institut, Helmholtz-Zentrum für Polar- und Meeresforschung, Bremerhaven, Germany; ^5^Department of Physiology and Pharmacology, Sapienza University of Rome, Rome, Italy; ^6^Radiobiology Unit, Belgian Nuclear Research Centre (SCKCEN), Mol, Belgium; ^7^Department of Molecular Biotechnology, Ghent University, Ghent, Belgium

**Keywords:** endocannabinoids, catecholamines, glucocorticoids, hypobaric hypoxia, high altitude, Antarctica

## Abstract

The Antarctic continent is an environment of extreme conditions. Only few research stations exist that are occupied throughout the year. The German station Neumayer III and the French-Italian Concordia station are such research platforms and human outposts. The seasonal shifts of complete daylight (summer) to complete darkness (winter) as well as massive changes in outside temperatures (down to -80°C at Concordia) during winter result in complete confinement of the crews from the outside world. In addition, the crew at Concordia is subjected to hypobaric hypoxia of ∼650 hPa as the station is situated at high altitude (3,233 m). We studied three expedition crews at Neumayer III (sea level) (*n* = 16) and two at Concordia (high altitude) (*n* = 15) to determine the effects of hypobaric hypoxia on hormonal/metabolic stress parameters [endocannabinoids (ECs), catecholamines, and glucocorticoids] and evaluated the psychological stress over a period of 11 months including winter confinement. In the *Neumayer III* (sea level) crew, EC and *n*-acylethanolamide (NAE) concentrations increased significantly already at the beginning of the deployment (*p* < 0.001) whereas catecholamines and cortisol remained unaffected. Over the year, ECs and NAEs stayed elevated and fluctuated before slowly decreasing till the end of the deployment. The classical stress hormones showed small increases in the last third of deployment. By contrast, at *Concordia* (high altitude), norepinephrine concentrations increased significantly at the beginning (*p* < 0.001) which was paralleled by low EC levels. Prior to the second half of deployment, norepinephrine declined constantly to end on a low plateau level, whereas then the EC concentrations increased significantly in this second period during the overwintering (*p* < 0.001). Psychometric data showed no significant changes in the crews at either station. These findings demonstrate that exposition of healthy humans to the physically challenging extreme environment of Antarctica (i) has a distinct modulating effect on stress responses. Additionally, (ii) acute high altitude/hypobaric hypoxia at the beginning seem to trigger catecholamine release that downregulates the EC response. These results (iii) are not associated with psychological stress.

## Introduction

Humans have an amazing ability to survive in extreme environments and to acclimatize to varying environmental conditions ranging from humid tropical forests to polar deserts (Burtscher et al., 2018; Ilardo and Nielsen, 2018). Stress hormones and other stress activated systems play an important role as mediators of acclimatization responses to changes in the environment (Dhabhar, 2018). To measure these hormones and evaluate the function of the corresponding and interacting physiological systems, helps to better understand such acclimatization processes and thus offers to explore options to prevent and counteract possible negative responses with detrimental effects on human physiological integrity.

It was demonstrated that catecholamine and cortisol responses to physical exercise differ under conditions of normoxia or hypoxia with higher concentrations under hypoxia (Mazzeo et al., 1994; Woods et al., 2017). However, the question whether normobaric and hypobaric hypoxia elicit the same reactions remains contradictory (Girard et al., 2012; Millet et al., 2012; Mounier and Brugniaux, 2012). Furthermore, to date, most studies investigated the consequences of acute or intermittent hypoxia (Xie et al., 2001; Calbet, 2003; Lusina et al., 2006; Sander, 2016) but data and knowledge about the effect of prolonged and chronic exposure to hypoxic conditions are rather scarce (Dhar et al., 2014).

Moreover, the exposure often compasses not only one environmental stressor (e.g., hypoxia) but demands the acclimatization to several interacting and combined stressors (e.g., hypobaric hypoxia and cold) (Burtscher et al., 2018). In this context, the acclimatization to one stressor was found to be able to modify and influence the response to the other, which is called cross-adaptation (Chauhan et al., 2015). It was evidenced for cold/heat and hypoxia (Launay et al., 2006; Lunt et al., 2010; Keramidas et al., 2015; Gibson et al., 2017) which modified their interaction but broad data in this field of research is still missing due to difficulties of the measure so that a general conclusion cannot be drawn to date. The same accounts for the acclimatization response of each individual that seems to be affected by distinct differences (Bartone et al., 2018).

Besides the sympathoadrenal and glucocorticoid system, the endocannabinoid system (ECS) plays an important role in coping with such stress reactions. Its lipid mediators the endocannabinoids (ECs) and chemically related *N*-acylethanolamides (NAEs) are very related with acclimatization processes at several physiological lines (e.g., psychological, metabolic, peripheral, and central nervous system) in response to environmental factors such as hypobaric hypoxia and temperature to reach physiological homeostasis (Campolongo et al., 2009, 2013; Richard et al., 2009; Chouker et al., 2010; Dlugos et al., 2012; Feuerecker et al., 2012; Hauer et al., 2013, 2014; Morena et al., 2014; Neumeister et al., 2015; Hanlon et al., 2016).

We investigated a cohort of healthy male individuals over 12 months including a 9-month overwintering period at two Antarctic research stations: Neumayer III near the Antarctic coast in the Queen Maud land and Concordia in Inner East Antarctica.

In general, the Antarctic environment is characterized by extreme shifts in daylight from 24 h light during the Antarctic summer to complete 24 h darkness during the winter period as well as massive changes in outside temperatures at onset of the Antarctic winter requiring complete confinement of the crew to the protective research stations. Thus, expeditioners suffer from sensory deprivation by a monotone environment with lack of stimuli, restricted food variation, and a distinct social narrowing with high potential for conflicts (Das et al., 2018). Furthermore, a disruptive circadian rhythm may influence the hormonal stress response (Chen et al., 2016; Vitale et al., 2018).

In detail, living conditions at Concordia are more severe and thus ensue more rigorous changes than at Neumayer III due to its inland location. Additionally, in contrast to Neumayer III which is situated at sea level, Concordia is situated at 3,233 m above sea level inducing an environment of hypobaric hypoxia to acclimatize to.

We hypothesized that (i) the extreme environmental conditions would lead to an increase of stress-related hormones and that (ii) this increase would be aggravated and sustained in the crew of Concordia Station as of their exposition to long-term, chronic hypobaric hypoxia.

The aim of our study was to investigate and precise the modulations of the stress-related ECS in humans exposed to harsh and extreme environmental living conditions as represented in Antarctica. The main focus hereby was set on the changes induced by chronic hypobaric hypoxia.

To confirm our hypothesis, we therefore determined hormonal/metabolic stress parameters (ECs, catecholamines, and glucocorticoids) and evaluated the psychological stress level in three expedition crews at Neumayer III (sea level) and in two at Concordia (high altitude), respectively.

## Materials and Methods

### Group of Study

In this prospective field study, in total, 15 healthy male participants were included for the *Concordia* (high altitude) crew investigation (seven individuals during the expedition period 2016 and eight volunteers in 2017) and 16 healthy male crew members for the *Neumayer III* (sea level) investigation (five individuals in 2013, six in 2014, and five in 2015). The crews at both stations changed in every expedition period so that every participant was included only once in the study. Demographic data of the participants are given in Table 1.

**Table 1:** Demographic data of participants of the respective campaign and Antarctic station at BDC.

	Crew 2016 Concordia (high altitude)	Crew 2017 Concordia (high altitude)	Total Concordia (high altitude)	Crew 2013 Neumayer III (sea level)	Crew 2014 Neumayer III (sea level)	Crew 2015 Neumayer III (sea level)	Total Neumayer III (sea level)
Number (*n*)	7	8	15	5	6	5	16
Age (years)	43.4 ± 4.6	35.3 ± 3.2	39.1 ± 2.8	33.8 ± 0.9	36.2 ± 3.7	43.4 ± 5.3	37.7 ± 2.3
Body mass index (kg/m^2^)	24.7 ± 0.6	24.8 ± 1.3	24.7 ± 0.8	27.37 ± 2.2	26.3 ± 1.0	27.1 ± 1.4	26.9 ± 0.8


*Data are mean ± SEM.*

### Concordia and Neumayer III Research Station

This study took place at two different Antarctic research stations: (i) the French-Italian inner-continental station *Concordia*, situated at 3,233 m above sea-level (high altitude; pressure level ∼640 to 650 hPa). It is located at a latitude/longitude of 75° 06′ S/123° 21′ E on an inland high ice plateau area called Dome C. The closest coastal region is approx. 1,100 km away (ii) the German coastal Antarctic station *Neumayer III* which is situated in the Atka Bay in the northeast Weddell sea on the Ekström shelf ice at sea level with the coordinates 70° 40′ S/8° 16′ W. Seasons in Antarctica are opposite to the northern hemisphere with Antarctic summer lasting from beginning of November to beginning of February and winter from May to August. The longest day (mid-summer) is in December and the shortest day in June (mid-winter). During the Antarctic summer the lack of a light/dark cycle results in 24 h of constant sunlight. During this period, average outside temperatures are around -50°C (Concordia, high altitude) and -3°C (Neumayer III, sea level). By contrast, during the winter season no sunlight is present and outside temperatures range around -60°C and can drop to -80°C at Concordia (high altitude) and -30°C at Neumayer III (sea level). In addition to the extreme outside temperatures, humidity is very low especially in inner Antarctica (Concordia, high altitude) leading to a very dry environment. Here, precipitation is very little throughout the year.

In order to maintain a regular circadian rhythm in this environment, a normal day–night cycle is tried to be respected by keeping regular hours for common meals, working duties, and evening activities.

In summary, these extreme conditions lead to a complete isolation (no access/exit possible) from the outer world during almost 9 months (mid-February to mid-October). Telecommunication with the outside world from the stations is possible *via* phone which can depend on current weather conditions. The same applies for internet access that is possible but not always reliable. All supply goods for the over-wintering period are stored in different areas inside the two stations, including different fridges and freezers (+4 to -25°C).

### Study Protocol

Data from two expedition campaigns at Concordia (high altitude) (2016 and 2017) and from three expedition campaigns at Neumayer III (sea level) (2013–2015) were analyzed. Data collection and blood sampling for stress hormone analyses in the study groups were performed on a monthly basis during the first week of the month and in the morning around 7:00–8:00 am starting in January/February after arrival of the crew members and continued through the whole year until October/November when the station was prepared for the next seasonal change of crew. One of the authors (CS and MF) was present at Concordia (high altitude) during December/January (summer season) of each campaign when the station was accessible by aircraft to help with the setup of the study protocol and for training of the crew to maximize study compliance. To establish procedural processing at Neumayer III (sea level) external assistance from the study team (AC) was present during the first Antarctic summer season. The training of the crew surgeons took place 1–3 months before deployment. During the actual overwintering period, data collection, blood sampling, and processing of the samples was performed by one of the crew surgeons (FvdB, CD, BF, HB, and PG-L) who have received several weeks of training in carrying out research protocols and in the long-term assessment and monitoring of the crew members during the winter period.

In addition to data collection and blood sampling in Antarctica, a baseline data collection (BDC) including blood sampling was performed in Europe. BDC took place approximately 2–4 months prior to departure to Antarctica and was performed close to sea level in Bremerhaven, Cologne, or Berlin, Germany.

This study was carried out in accordance with the recommendations of the local Ethics Committee of the University of Munich with written informed consent from all subjects. All subjects gave written informed consent in accordance with the Declaration of Helsinki. The protocol was approved by the local Ethics Committee of the University of Munich [Protocols# 332-08, 524-15]. All samples were collected after an 8 h fasting period in the morning around 7:00–8:00 am. Physical exercise was not allowed for 24 h prior to sample collection.

### Biochemical Measurements

Biochemical measurements were performed from blood samples taken in EDTA tubes for blood cell count, in lithium-heparinized tubes (S-Monovette^®^, Sarstedt, Nümbrecht, Germany) for EC and NAEs measurements, from saliva (Salivette^®^, Sarstedt, Nümbrecht, Germany) for the determination of free cortisol and from 12 h pooled urine [from 19:00 to 7:00 (nighttime)] for catecholamine analyses.

All samples were taken in the early morning and after an overnight fasting period. Blood samples for blood cell count were stored at ambient temperature until further processing the same day. Blood samples drawn for ECs were immediately placed on ice water to prevent temperature effects (Vogeser et al., 2006). Blood, urine, and saliva samples were frozen immediately after processing and stored throughout the expedition year at a minimum temperature of at least -25°C. The return of the frozen samples from Concordia (high altitude) and Neumayer III (sea level) back to Germany was effected by ship and plane with a strict temperature monitoring assuring a temperature of at least -25°C or colder. Samples were stored at -60°C (freezer) till further processing and analyses after arrival at Munich.

#### Blood Cell Count

EDTA-anti-coagulated blood samples were used to determine the blood cell count using the on-site QBC Autoread plus automated analyzing system (QBC Diagnostics, Port Matilda, PA, United States). Hemoglobin concentration and hematocrit were quantified.

#### Endocannabinoid and NAEs Measurements

After sampling, blood samples were immediately centrifuged, the plasma transferred into Eppendorf tubes and frozen at -60°C without any delay (Hauer et al., 2013). This procedure allows the storage of EC samples for at least 6 months (Di Marzo et al., 2009).

Plasma concentrations of the ECs anandamide (AEA), 2-arachidonoylglycerol (2-AG) as well as the *N*-acyl-ethanolamides (NAEs) palmitoylethanolamide (PEA), oleoylethanolamide (OEA), and stearoylethanolamide (SEA) were determined using a LC-High Resolution LC-MS/MS instrument combination (Agilent 1290/Sciex Triple TOF 6600) in positive high-resolution MRM (multiple reaction monitoring, MS resolution ∼16,000) mode. Chromatographic separation was achieved on a RP-C18 column (Zorbax C8, 2.1 mm × 50 mm, 3.5 μm; Agilent). Both components of the binary gradient contained a mixture of 2 mmol aqueous ammonium acetate and ACN at a mixing ratio of 95+5 (A) and 5+98 (B). Both buffers were moderately acidified with 0.1% acetic acid. The separation gradient started at an amount of 10% B which was linearly increased to 100% B at 10 min and was finally kept constant for another 2 min at a flow rate of 250 μl/min.

#### Urine Catecholamines

The catecholamines norepinephrine and epinephrine were determined from pooled nighttime urine samples reflecting the required conditions of physical inactivity. Nighttime samples were used to obtain baseline secretion of catecholamines in contrast to daytime urine samples which reflect physical challenges and activity throughout the awake phase.

Samples were stored at -60°C till final processing in Europe. After defrosting, quantification of catecholamine concentrations was performed at the Institute of Clinical Chemistry, University of Munich, Germany, using HPLC (Chromsystems, Martinsried, Germany). In order to determine the absolute mass of excreted catecholamines, urine catecholamine concentrations were multiplied by respective urine volume.

#### Saliva Cortisol

Until final measurement, samples were stored frozen at -60°C. Cortisol concentrations were quantified by an electrochemiluminescence immunoassay (Elecsys 2010, Roche, Mannheim, Germany) at the Institute of Clinical Chemistry, University of Munich, Germany.

### Psychological Evaluation and Measurements

Two different paper questionnaires were performed to quantify and analyze each participant’s emotional stress level:

#### CST (Short Questionnaire on Current Stress)

The test is validated to reflect the level of acute emotional stress (Current Stress Test, CST) (Müller and Basler, 1993). It consists of six questions and is highly sensitive to acute situational changes in subjective stress experience. Six items of paired positive and negative adjectives referring to perceptions of current stress and strain or relaxation (e.g., “tense–calm,” “uneasy–relaxed”) must be rated on a six-point Likert scale. The range for total item means is 1–6, with higher values indicating an increased stress experience. The composition of the questionnaire entails that the subject usually does not remember the previous ratings, thus preventing carry-over effects.

#### Spielberger State Trait Anxiety Inventory (STAI)

It consists of two parts (each 20 questions) rating the answers on a 4-point scale. The score for the global test may range between 40 and 160 points. It evaluates state anxiety (in a specific situation) or trait anxiety (as part of a person’s character) (Spielberger et al., 1970).

### Statistical Analyses

Normal distribution of sample data was tested using the Shapiro–Wilk test. Within group comparisons (e.g., changes in EC levels over time) were performed by one way repeated measures analysis of variances (one-way RM-ANOVA) followed by a *post hoc* Holm–Sidak test to determine which measurements were significantly different and to correct for multiple comparisons. Between-group comparisons were performed using a *t*-test for normally distributed data and the Mann–Whitney *U* test for non-parametric data followed by a Bonferroni correction for multiple comparisons, respectively. For all testing, a *p*-value <0.05 was regarded as statistically significant. Data are displayed as mean ± SEM. Statistical calculations were performed using SigmaPlot^®^ (Systat, Software, Chicago, IL, United States) and IBM SPSS Statistics v24, United States.

## Results

### Biochemical Measurements

#### Blood Cell Count (Table 2)

##### Neumayer III (sea level)

Hemoglobin concentrations were moderately elevated during the isolation period in February, March, and June to October when compared to BDC (15.4 ± 0.18 g/dl, *p* < 0.05) but still fluctuated in the normal range. The hematocrit always stayed on a normal level.

**Table 2:** Hemoglobin concentrations (g/dl) and hematocrit (%).

	Neumayer III (sea level)	Concordia (high altitude)	Neumayer III (sea level)	Concordia (high altitude)
	Hb (g/dl)	Hb (g/dl)	Hct (%)	Hct (%)
BDC	15.4 ± 0.18	14.4 ± 0.35	47 ± 0.6	42 ± 1#
February	16.4 ± 0.33*	17.9 ± 0.28*,#	49 ± 0.6	52 ± 1.2*
March	16.6 ± 0.24*	18.0 ± 0.24*,#	49 ± 0.6	51 ± 2.1*
April	16.1 ± 0.39	17.9 ± 0.20*,#	48 ± 1.1	53 ± 2*
May	16.2 ± 0.23	17.8 ± 0.20*,#	49 ± 0.6	53 ± 0.6*,#
June	16.3 ± 0.19*	17.5 ± 0.17*,#	49 ± 0.5	51 ± 0.8*
July	16.5 ± 0.18*	17.7 ± 0.33*	49 ± 0.5	53 ± 1.1*
August	16.6 ± 0.23*	18.0 ± 0.27*,#	50 ± 0.7	53 ± 0.8*
September	16.6 ± 0.36*	17.7 ± 0.22*	50 ± 0.8	52 ± 0.9*
October	16.3 ± 0.26*	18.4 ± 0.19*,#	49 ± 0.7	53 ± 0.6*,#
November	16.1 ± 0.30	18.5 ± 0.28*,#	48 ± 0.6	54 ± 1*,#


*Data are mean ± SEM. Hb, hemoglobin; Hct, hematocrit; BDC, baseline data collection. ^∗^Significant difference to BDC. ^#^Significant difference between Neumayer III (sea level) and Concordia (high altitude).*

##### Concordia (high altitude)

Hemoglobin concentrations and hematocrit were significantly elevated in every month throughout the deployment compared to BDC with highest values at the end of the stay (Hb BDC 14.4 ± 0.35 g/dl to November 18.5 ± 0.28 g/dl; *p* < 0.001 versus Hct BDC 0.42 ± 0.01 to November 0.54 ± 0.01; *p* < 0.001).

##### Neumayer (sea level) versus Concordia (high altitude)

Hemoglobin concentrations were significantly higher in the Concordia (high altitude) crew from February to June, in August, October, and November (*p* < 0.05). Hematocrit values were significantly elevated in the crew at Concordia (high altitude) compared to the crew at Neumayer III (sea level) in May and at the end of the stay in October and November (*p* < 0.001).

#### Endocannabinoid (EC) and NAEs Plasma Concentrations (Figures 1A–E)

At BDC in Europe before deployment of the crews to Antarctica, EC and NAEs values were within the normal range in both crews.

**FIGURE 1:**
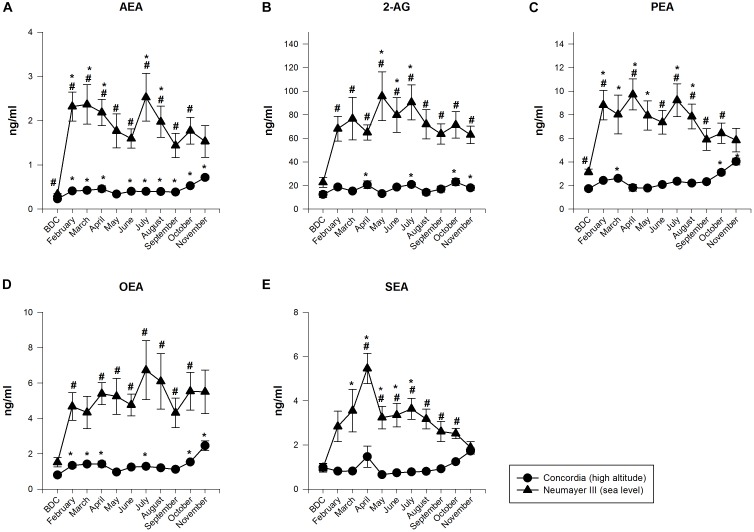
Endocannabinoid (EC) and *N*-acylethanolamide plasma concentrations at Neumayer III (sea level) (*n* = 7–15) and Concordia (high altitude) (*n* = 15–16); data are means ± SEM; units are ng/ml; BDC, baseline data collection; AEA, anandamide **(A)**; 2-AG, 2-arachidonoylglycerol **(B)**; PEA, palmitoylethanolamide **(C)**; OEA, oleoylethanolamide **(D)**; SEA, stearoylethanolamide **(E)**; ^∗^significant difference to BDC; ^#^significant difference between Neumayer III (sea level) and Concordia (high altitude) crews.

##### Neumayer III (sea level)

The NAEs and ECs (AEA and 2-AG) increased their concentrations significantly up to 10-fold from BDC already at the beginning of the deployment, fluctuated throughout the year but always on a highly elevated level compared to BDC, and finally decreased slowly till the end of the deployment but not returning to BDC values [*AEA* increased from BDC 0.34 ± 0.02 to 2.53 ± 0.54 ng/ml; *p* < 0.001 (July). *2-AG* from BDC 22.7 ± 4.23 to 95.74 ± 20.71 ng/ml; *p* < 0.001 (May). *PEA* from BDC 3.15 ± 0.25 to 9.72 ± 1.31 ng/ml; *p* < 0.001 (April). *OEA* from BDC 1.52 ± 0.26 to 6.73 ± 1.67 ng/ml, no significance (July). *SEA* from BDC 0.97 ± 0.2 to 5.46 ± 0.68 ng/ml; *p* < 0.001 (April)].

##### Concordia (high altitude)

The NAEs and ECs (AEA and 2-AG) fluctuated on a constantly low level with intermittent small significant increases. Significant increases were found at the end of the deployment [*AEA* increased from BDC 0.23 ± 0.01 to 0.72 ± 0.04 ng/ml; *p* < 0.001 (November). *2-AG* from BDC 12.43 ± 2.65 to 23.04 ± 3.09 ng/ml; *p* < 0.001 (October). *PEA* from BDC 1.73 ± 0.18 to 4.03 ± 0.29 ng/ml; *p* < 0.001 (November). *OEA* from BDC 0.8 ± 0.12 to 2.46 ± 0.28 ng/ml; *p* < 0.001 (November). *SEA* from BDC 0.97 ± 0.17 to 1.72 ± 0.14 ng/ml; no significance (November)].

##### Neumayer III (sea level) versus Concordia (high altitude)

The differences in NAEs and EC (AEA and 2-AG) values between the crews at Neumayer III (sea level) and Concordia (high altitude) were highly significant for all neurotransmitters and nearly throughout the whole isolation period and duration of stay in Antarctica (*AEA* June *p* < 0.05, for all other months except the end of the stay *p* < 0.001. *2-AG* for all months *p* < 0.001 except BDC. *PEA* October *p* < 0.05, for all other months except March, May, and the end of the stay *p* < 0.001. *OEA* May and June *p* < 0.05, for all other months except BDC, March, and November *p* < 0.001. *SEA* June *p* < 0.05, for all other months except BDC, February, March, and November *p* < 0.001). At the end of the stay, EC and NAEs scores of the crews at both stations converged since their concentrations at Neumayer III (sea level) decreased but increased at Concordia (high altitude).

### Urinary Catecholamine Excretion

#### Norepinephrine (Figure 2A)

##### Neumayer III (sea level)

Norepinephrine levels during the night stayed on a consistent level throughout the confinement and showed no significant changes. Only toward the end of the months of August and September, the levels raised albeit not significantly.

**FIGURE 2:**
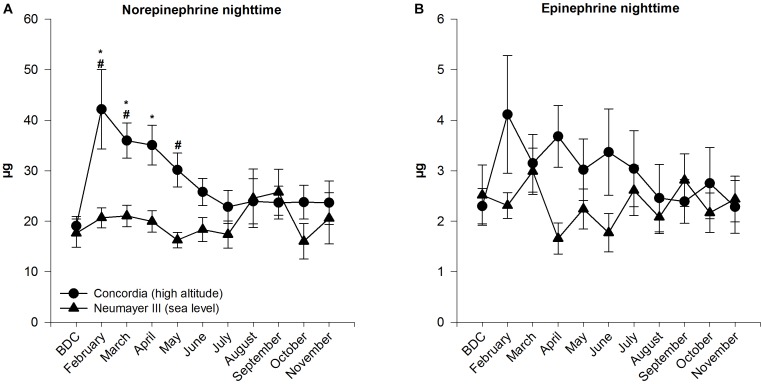
Norepinephrine **(A)** and epinephrine **(B)** in 12-h nighttime urine at Neumayer III (sea level) (*n* = 11–16) and Concordia (high altitude) (*n* = 11–15); data are means ± SEM; units are μg (collection time 12 h); BDC, baseline data collection; ^∗^significant difference to BDC; ^#^significant difference between Neumayer III (sea level) and Concordia (high altitude) crews.

##### Concordia (high altitude)

Nighttime norepinephrine amount increased significantly from BDC to February (BDC 19.07 ± 1.88 μg to February 42.18 ± 7.87 μg; *p* < 0.001) and stayed on a significantly elevated high level until April. Peak values were already reached in February before levels decreased slowly and constantly till July. In July, norepinephrine mass plateaued until the end of the stay at levels similar to BDC.

##### Neumayer III (sea level) versus Concordia (high altitude)

A significant difference between the nighttime norepinephrine masses in the crews of the two stations was found in February, March, and May (*p* ≤ 0.001 to *p* < 0.05). The mass of excreted norepinephrine was constantly higher in the Concordia (high altitude) crew except for the months August and September.

Calculated SEMs were higher in the Concordia (high altitude) than in the Neumayer III (sea level) crew and exceeded at the beginning of the expedition when significant differences between both stations were stated.

#### Epinephrine (Figure 2B)

##### Neumayer III (sea level)

In the Neumayer III (sea level) crew, epinephrine levels during the night varied inconsistently during the stay and ended at almost the same level as at BDC.

##### Concordia (high altitude)

Epinephrine excretion increased in February (from BDC 2.30 ± 0.35 to 4.11 ± 1.17 μg), although without statistical significance and decreased slowly over the year with little fluctuations to finally reach BDC levels at the end of the year.

##### Neumayer III (sea level) versus Concordia (high altitude)

Epinephrine masses started in both crews at the same level. During the year, levels were always higher at Concordia (high altitude) except at the end in the month September but showed no significant differences between the two crews.

### Cortisol in Saliva

#### Morning Cortisol (Figure 3)

##### Neumayer III (sea level)

Morning cortisol concentrations increased slightly in the first 3 months but without statistical significance and then fluctuated around the baseline level.

**FIGURE 3:**
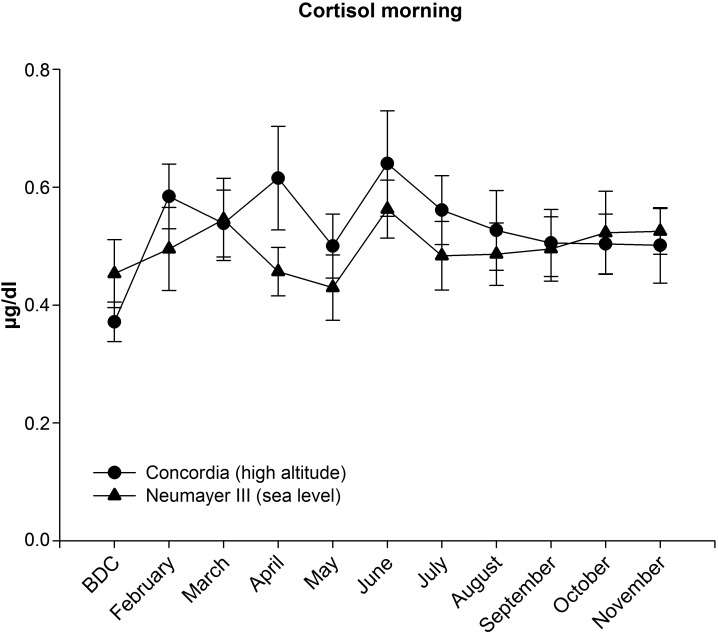
Cortisol in saliva in the morning at Neumayer III (sea level) (*n* = 13–16) and Concordia (high altitude) (*n* = 12–15); data are means ± SEM; units are μg/dl; BDC, baseline data collection.

##### Concordia (high altitude)

Morning cortisol concentrations increased until April albeit not significantly then dropped in May before reaching the maximum level in June (from BDC 0.37 ± 0.03 to 0.64 ± 0.09 μg/dl). Afterward, cortisol declined constantly but stayed at a level higher than at BDC.

##### Neumayer III (sea level) versus Concordia (high altitude)

During the deployment, cortisol concentrations were nearly constantly higher in the Concordia (high altitude) crew but without any significant differences between the two crews.

#### Evening Cortisol (Data Not Shown)

Evening cortisol concentrations were constantly lower than in the morning and did not exceed normal values at either station (Neumayer III (sea level) between 0.12 and 0.22 μg/dl; Concordia (high altitude) between 0.13 and 0.17 μg/dl). At Concordia (high altitude), values ranged minimally higher than at Neumayer III (sea level) during the deployment except at the end of the stay (August-November). The circadian rhythm was maintained.

### Psychological Evaluation and Measurements

#### CST (Short Questionnaire on Current Stress) (Table 3)

##### Neumayer III (sea level)

The acute stress test (CST) revealed no significant changes throughout the deployment period. Mean CST values in the morning ranged between 2.2 ± 0.3 and 2.7 ± 0.4 points. Evening results displayed the same pattern (data not shown).

**Table 3:** Psychometric data of CST.

	CST m Neumayer III (sea level)	CST m Concordia (high altitude)
BDC	2.4 ± 0.2	1.9 ± 0.2
February	2.7 ± 0.2	2.0 ± 0.1
March	2.3 ± 0.1	1.9 ± 0.1
April	2.7 ± 0.3	2.0 ± 0.2
May	2.2 ± 0.3	1.9 ± 0.1
June	2.4 ± 0.3	2.0 ± 0.2
July	2.4 ± 0.2	1.7 ± 0.1
August	2.3 ± 0.3	1.9 ± 0.2
September	2.3 ± 0.2	1.9 ± 0.1
October	2.4 ± 0.3	1.9 ± 0.1
November	2.7 ± 0.4	1.8 ± 0.2


*Data are mean ± SEM. CST, Current Stress Test; BDC, baseline data collection.*

##### Concordia (high altitude)

No significant changes throughout the deployment period were detectable, with mean CST values ranging in the morning between 1.7 ± 0.1 and 2.0 ± 0.1 points. The same results were measured in the evening (data not shown).

##### Neumayer III (sea level) versus Concordia (high altitude)

CST scores were consistently higher at Neumayer III (sea level) than at Concordia (high altitude) independent of the time point and the time during the day but without any significant differences between the two crews (evening data not shown).

#### Spielberger State Trait Anxiety Inventor (STAI) (Table 4)

##### Neumayer III (sea level)

Mean state evaluation scores ranged from 32.4 ± 2.5 to 38.5 ± 3.4 points whereas the trait assessment showed mean values between 32.3 ± 2.5 and 35.5 ± 3.4 points. No significant changes were stated.

**Table 4:** Psychometric data of STAI.

	STAI state Neumayer III (sea level)	STAI state Concordia (high altitude)	STAI trait Neumayer III (sea level)	STAI trait Concordia (high altitude)
BDC	32.4 ± 2.5	28.8 ± 2.0	33.8 ± 2.0	28.4 ± 1.0
February	36.4 ± 2.4	28.6 ± 1.4	32.6 ± 2.3	29.9 ± 1.5
July	34.9 ± 2.3	27.3 ± 1.3	32.3 ± 2.5	29.4 ± 2.0
November	38.5 ± 3.4	30.4 ± 2.8	35.5 ± 3.4	29.6 ± 1.3


*Data are mean ± SEM. STAI, Spielberger State Trait Anxiety Inventory; BDC, baseline data collection.*

##### Concordia (high altitude)

State mean scores at Concordia (high altitude) ranged from 27.3 ± 1.3 to 30.4 ± 2.8 points; trait means ranged between 28.4 ± 1.0 and 29.9 ± 1.5 points. These findings were not statistically significant.

##### Neumayer III (sea level) versus Concordia (high altitude)

Between the two crews, the anxiety level (state and trait) was consistently higher at Neumayer III (sea level) but without any significant differences.

## Discussion

The present study investigated humans during long-term acclimatization to the highly aversive environment of two Antarctic research stations. Main focus of the research was to investigate the effects of hypobaric hypoxia on stress-related metabolites. Interestingly and contrary to expectations, a massive increase of these metabolites was observed under normoxic conditions (Neumayer III, sea level) while concentrations under hypobaric hypoxia (Concordia, high altitude) stayed low during the isolation period but enhanced significantly at the end. Conversely, catecholamines showed an increase at the beginning of exposure to high altitude being prolonged for norepinephrine whereas cortisol showed no changes.

It seems that the exposure of humans from a physiologically familiar to an unknown and physically very challenging environment triggers this enhanced EC response and that hypobaric hypoxia modulates this response in terms of a downregulation. Downregulated expression or lack of increased expression of ECs in the Concordia (high altitude) crews may originate in hypoxia signaling and EC metabolism. In the tumor microenvironment, hypoxia is known to upregulate the expression of cyclooxygenase-2 (COX-2) *via* hypoxia-inducible factor-1 (HIF-1) signaling (Greenhough et al., 2009). COX-2 is one of the enzymes responsible for metabolism of ECs (Sugimoto et al., 2017) with the main substrates being AEA and 2-AG (Chiurchiu et al., 2015). Therefore, missing upregulation of ECs at Concordia (high altitude) might be due to hypoxia-induced upregulation of COX-2 (and possibly other enzymes) which in turn oxidizes ECs into compounds (e.g., prostaglandin-ethanolamides, hydroxy-AEAs) (Chiurchiu et al., 2015) that were beyond the scope of our investigations. Furthermore, other dysregulations of degrading and metabolizing systems of the ECs [e.g., fatty acid amide hydrolase (FAAH), intracellular transporters] that were not focus of our study might also have contributed to the stated downregulation of the ECS.

However, HIF-1α expression under long-term hypobaric hypoxia seems to be time-dependent. A short-lived transient activation of HIF-1α-dependent pathways was observed at the beginning of exposition to high altitude (Bigham and Lee, 2014; Bigham, 2016) but seems to elapse gradually over time (Petousi et al., 2014). Thus, this regulation pathway seems to be less significant in acclimatization processes upon long-term exposition to hypobaric hypoxia (Baze et al., 2010). This is in good accordance with findings of Feuerecker et al. (2018) who observed in a former study at Concordia a downregulation of HIF-1α expression under chronic hypobaric hypoxia. Goyal and Longo (2014) observed similar results albeit in an animal model. Against the background of the assumed COX-2 influence on EC metabolism, one might speculate that this mechanism might have also contributed to the rising EC levels at Concordia (high altitude) at the end.

The hypothesis that an unknown and physically challenging environment triggers enhanced EC responses is in good accordance with recent studies which demonstrated that the consequences of strenuous physical conditions (e.g., sleep restrictions, physical exercise) are able to induce a heightened EC response (Cedernaes et al., 2016; Hanlon et al., 2016).

In the light of such stressful conditions, we also expected enhanced catecholamines and cortisol levels due to an elevated sympathetic tone but surprisingly the stress hormone answers stayed normal in the Neumayer III (sea level) expeditioners. One possible explanation for these unexpected observations derives from the interaction between the EC and the sympathetic nervous system and the glucocorticoids. During an acute stress model realized by parabolic flights, subjects who experienced no motion sickness showed similar results to ours presenting high EC concentrations and simultaneously low cortisol levels. By contrast, in subjects with motion sickness, the hormones’ balance was inversed (Chouker et al., 2010). Increasing EC levels were also described in a chronic stress model when examining astronauts on the International Space Station (Strewe et al., 2012).

On the other hand, Yi et al. (2016) reported during a 520 days lasting confinement in the course of a simulated mission to Mars a reduction in circulating 2-AG but a significant increase in urinary norepinephrine and saliva cortisol. The chronic stressor of confinement resulted here in an interaction of these systems that is in contrast to our findings albeit it also displays its apparent inverse character. Moreover, the hypothesis of an inverse interaction is also supported by Ishac et al. (1996) who stated an inhibitory effect of presynaptic cannabinoid CB_1_ receptors on noradrenaline release in peripheral sympathetic nerves and Surkin et al. (2018) who evidenced that pharmacological augmentation of EC signaling reduces the neuroendocrine response to stress. Niederhoffer and others also found this inhibitory effect of ECs on noradrenaline to be the cause of cardiovascular depression (Wagner et al., 1998; Niederhoffer et al., 2003; Pfitzer et al., 2005). However, opposite reactions were also described dependent on the acting EC (Kurihara et al., 2001). Furthermore, the way of interaction seems to vary dependent on the physiological system that is examined. Indeed, Simkins et al. (2016) described a positive correlation when they detected a reduced noradrenergic signaling in the spleen capsule in the absence of cannabinoid receptors. Additionally, cannabinoid receptor agonists were shown to induce peripheral antinociception by activating the noradrenergic system (Romero et al., 2013) and they increased norepinephrine efflux in the frontal cortex in an animal model (Page et al., 2008).

By contrast, when analyzing the data of the Concordia (high altitude) crew, we stated that low EC levels were associated with significantly increased norepinephrine concentrations and increased cortisol levels albeit not significant. A former study of our research group in the same Antarctic environment already evidenced similar results with elevated catecholamine levels and assumed their association with stated immune alterations (Feuerecker et al., 2014). Furthermore, several other studies in (simulated) high altitude investigated well the impact of hypobaric hypoxia on sympathoadrenal and adrenocortical stress responses and found a positive correlation (Calbet, 2003; Simeoni et al., 2011; Dhar et al., 2014; Aliyev et al., 2017; Woods et al., 2017). Woods et al. (2017) showed that simulated altitude under normobaric or hypobaric hypoxia (equivalent to 3,375 m) appears to induce similar effects in humans than genuine high altitude. Nevertheless, these effects of hypoxia are also variable when combined with other co-(stress) factors such as, e.g., immobilization in bed rest. Thus, we demonstrated in a former study exposing subjects to 21 days of normobaric hypoxia (simulated altitude ∼ 4,000 m; 14% O_2_) and/or bed rest a reduced sympathoadrenal answer after exposition to this combination of stressors. Normobaric hypoxia alone did not induce any significant changes in the catecholamine or cortisol answer (Strewe et al., 2017). However, the results of this study suggest a different mechanism of regulation due to the hypobaric hypoxic environment at Concordia. This mechanism seems to be based on an inverse interaction of the sympathoadrenal with the EC response. Hypobaric hypoxia would therefore either downregulate the EC response or upregulate the sympathoadrenal answer with subsequently raised norepinephrine levels which, in turn, would downregulate and suppress the other system, respectively. The opposite action could be stated in the Neumayer III (sea level) crew.

The hypothesis of an upregulated sympathoadrenal answer is supported by the findings while the mission approaches its end. In the course of time, the sympathoadrenal response declined gradually and stagnated on a low level at the end. Here, interestingly, the EC response started to increase and concentrations of, e.g., AEA and 2-AG were significantly higher than at BDC.

The increase of ECs and NAEs at Concordia (high altitude) at the end with the adjustment of the physiological answers is in line with the hypothesis of a recent study reported by Alarcon-Yaquetto et al. (2017) who demonstrated significant higher NAE concentrations in natives of high altitude (3,830 m) with higher hemoglobin concentration and lower pulse oxygen saturation. Apparently, the time course and the long-term impact of the physical conditions seem to play an important role in the development of the physiological answer. In addition, Feuerecker et al. (2012) evidenced no changes in the human ECS under short-term hypobaric hypoxia (altitude of 3,196 m) but only in its combination with physical effort. The opposite effect of an enhanced EC response was probably due to the study design that displayed an acute stress model. Furthermore, Strewe et al. (2017) found no changes in the ECS when exposing humans for 21 days to normobaric hypoxia (14% O_2_ and a simulated altitude of ∼4000 m) either in combination with bed rest or in an ambulatory setting. Here, the different results may be explained by the different physical stressors and the shorter time frame. Furthermore, differences in reaction and regulation patterns of the ECS may also vary dependent of the status and type of cell that is examined (tumor versus healthy cells as mentioned above).

Additionally, in the Concordia (high altitude) crew, great SEMs for norepinephrine (especially in the first months of exposition to hypobaric hypoxia), epinephrine, and cortisol displayed a great inter-individual variability in the concentrations measured that was not found in the Neumayer III (sea level) crew. This might express the individual responses of acclimatization to high altitude in the subjects (Siques et al., 2009; Hermand et al., 2015; Richalet and Lhuissier, 2015).

In summary, our findings at both Antarctic stations evidence that human exposure to a physically challenging and physiologically new and unknown environment with extreme conditions generates a neuroendocrine reaction pattern of interacting physiological systems. Herein, high altitude exposure evokes a significant increase of norepinephrine at the beginning in contrast to other neuroendocrine parameters. Furthermore, there seems to be a great variability in the inter-individual acclimatization responses to high altitude.

As seen by the results in contrast to the baseline data, the reaction pattern seems to display and express a long-term acclimatization process of the human body and physiology to these conditions rather than a pathological process as its intensity and efficiency diminishes over time to finally return to a nearly “normal” status.

Furthermore, our results attribute and relate these reactions to the physical challenge in this environment and do not support an explanation based on increased psychological stress. As all inquired stress questionnaires on both stations negated its presence. However, as a limiting factor, it must be considered that all participants in such expeditions are highly motivated and well prepared which might be a reason for the absence of a distinct psychological effect. Furthermore, such psychological evaluation tests depend on the motivation and honesty (manifested self-reflection) of the subject. If one wants to mask his real feelings and psychological state of mind, any emotional impairment might just be negated. Moreover, a constant higher score level in these tests in the Neumayer III (sea level) crew, albeit in the normal range, might also just display national differences in carrying out the tests or depend on the way how the tests were explained to the subjects in the first place.

### Limitations

The Neumayer III crew served as control group at sea level to identify the delta effects of hypobaric hypoxia compared to the crew at Concordia. This is not an ideal control group as (i) groups differ in their demographics (BMI) and (ii) not only hypoxia varies but also all other environmental conditions are alleviated at the Antarctic coast (e.g., temperatures, wild life, etc.) compared to Concordia located in inner Antarctica. A better control would have been either a cross-over study design where the same subjects would have been exposed to both, low and high altitude with the same light exposure time, same food, seasonal variations, and duration of expedition or a longitudinal study design with the same subjects. However, even in these two design models, either the same study year (cross-over design) or the different environmental conditions (low versus high altitude) would not be applied to the respective investigated subjects. Moreover, both designs seem however not feasible in this environment from many perspectives (participants, logistics, costs, and others).

Additionally, daily physical activity and challenge of the participants as well as their physical conditions and fitness might interfere with the measures taken so that in an ideal setting their monitoring would be warranted. Moreover, an investigation of each individuals’ peculiar physiological response could be of interest and importance to define personal differences and potentially identify corresponding groups with similar reaction patterns.

Furthermore, even though Antarctic climate only changes slowly due to global climate change and environmental conditions at both stations stay relatively stable from 1 year to the other, natural undulations in temperature, humidity, day–night cycle, etc. exist, and thus, a possible influence on the measures made cannot fully be excluded.

## Data Availability

The raw data supporting the conclusions of this manuscript will be made available by the authors, without undue reservation, to any qualified researcher.

## Author Contributions

MF, AC, and GS designed the work. CS, MF, AC, CD, FvdB, BF, HB, and PG-L collected the data. All authors (CS, DT, CD, BF, FvdB, HB, AS, PG-L, PC, DM, RQ, MM, SB, EK, GS, AC, and MF) performed data analysis and interpretation as well as drafting the article and critical revision for important intellectual content. All authors gave final approval of the version to be submitted and any revised version.

## Conflict of Interest Statement

The authors declare that the research was conducted in the absence of any commercial or financial relationships that could be construed as a potential conflict of interest.
